# Recent Advances in MXene-Based Screen-Printed Electrochemical Sensors for Point-of-Care Biomarker Detections

**DOI:** 10.3390/bios15120804

**Published:** 2025-12-08

**Authors:** Thao Thi Nguyen, Liang Zhou, Jinming Kong, Aiqin Luo, Zikai Hao, Jiangjiang Zhang

**Affiliations:** 1 Key Laboratory of Molecular Medicine and Biotherapy, Ministry of Industry and Information Technology, School of Life Science, Beijing Institute of Technology, Beijing 100081, China; thaonguyenhus96@gmail.com (T.T.N.); bitluo@bit.edu.cn (A.L.); 2 School of Environmental and Biological Engineering, Nanjing University of Science and Technology, Nanjing 210094, China; liangzh@njust.edu.cn (L.Z.); j.kong@njust.edu.cn (J.K.)

**Keywords:** screen-printed electrochemical sensors, Mxenes, point-of-care tests, biomedical applications

## Abstract

Contemporary biomedical diagnostics increasingly demand high sensitivity for pathogen detection and real-time health monitoring. In response to these requirements, screen-printed electrochemical sensors (SPEs) have emerged as a practical analytical platform owing to their low cost, portability, and compatibility with point-of-care and wearable systems. In the recent past, nanomaterials in two-dimensional format, especially MXenes, have gained much interest due to their high electrical conductivity, controllable surface chemistry, and biocompatibility, which can improve the performance and applicability of SPEs. The current review concentrates on the latest developments between 2020 and 2025, providing a critical assessment of research employing MXene-based nanomaterials for the modification and development of screen-printed electrode platforms. We provide an overview of fabrication techniques, printing methods, and surface modification methods, and proceed with an analysis of the electrochemical performance of MXenes and MXene-based heterostructures. Lastly, contemporary issues are considered, and opinions are suggested to facilitate the translation of MXene-functionalized SPEs to real biomedical diagnosis solutions.

## 1. Introduction

Electrochemical sensors constitute a critical analytical tool in biomedical research and clinical applications, particularly for the detection of cancer biomarkers, pathogenic agents, metabolic by-products, and signaling molecules. Current research in this field increasingly focuses on electrode miniaturization and the development of printed sensor platforms tailored for point-of-care (POC) diagnostics. These electrochemical sensors on paper make it possible to integrate elements of biorecognition with electrochemical transducers, and thus, real-time or near-real-time detection of target analytes, even in complicated biological samples. Specifically, one of the key technologies to support the future generation of electrochemical sensors is the printed electron–chemical technology, which is flexible, has a low-cost of production, and could be scaled to mass production. Inkjet printing, screen printing, offset printing, and stencil printing are all printing methods that enable the creation of sensors on flexible materials, like textile materials, paper, and plastics. Such approaches not only make the manufacturing process easier but also improve the compatibility and integration of sensors with electronic systems [1,2,3,4].

Printed electrochemical sensors have a high level of integrability and compactness, which means that they have a high potential of coming up with portable and easy-to-use apparatus. The systems are especially ideal for being implemented in resource-constrained environments such as home care and remote locations, and significantly lessening dependence on central laboratory facilities. Practically, screen-printed electrodes (SPEs) have been successfully used in different POC diagnostics, such as the fast detection of SARS-CoV-2 antigens [5], emergency screening of cardiac biomarkers [6], and glucose monitoring in diabetes control [7]. The most recent developments of the functional nanomaterials have contributed to solving the sensor performance significantly, with several systems already being explored and put into practice including metal nanoparticles (AuNPs, PtNPs, AgNPs), metal oxide nanoprecursors, quantum dyes, carbon nanotubes and nanofibers, graphene and its derivatives, conducting polymers, covalent/metal–organic frameworks (COFs/MOFs) [8], ionic liquids, and transition metal dichalcogenides (TMDs). These nanomaterials with sensing applications, MXenes in particular, have received specific consideration due to their distinctive layered geometry and bandgap tunability that significantly increases the sensitivity of the sensor and enhances the signal-to-noise ratio (SNR) [9].

A growing body of research has demonstrated the outstanding performance of MXene-based architectures in advanced electrochemical sensors. For example, a nanoengineered three-dimensional hybrid hydrogel has been utilized as an antifouling coating to improve the electrochemical aptasensing of estradiol [10]. Another study using a smartphone as a platform with a built-in, portable electrochemical sensor (ip-ECS) that uses gold nanoparticles (AuNPs) [11] and MXene-modified SPEs allowed the detection of cortisol in the sweat at the site [12]. Such sensor designs not only underscore the potential impressive outlook of MXenes in the overall improvement of the electrochemical sensing performance but also reflect the general direction of development of the next-generation diagnostic devices that can provide quick, precise, and economical analysis to use in healthcare diagnostic procedures, environmental measurements, and manufacturing processes. In line with this, this review will be focused on a summary of the recent development in MXene printed electrochemical sensors when applied in POC applications. Together with the consideration of the structure–performance relationships, the review is concerned with the contemporary challenges and perspectives of the evolution of the printed sensors in the setting of the digital healthcare integration, which ultimately leads to the achievement of smart, personalized, and accessible diagnostic platforms.

## 2. MXene Materials and Their Electrochemical Properties

**Figure 1:**
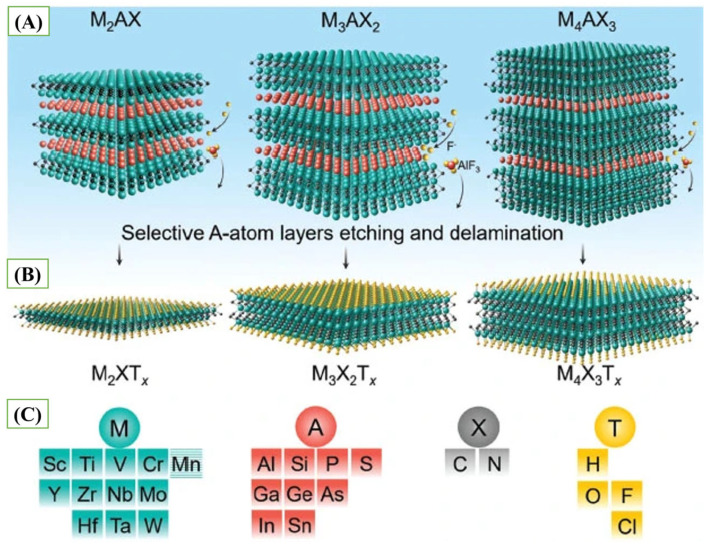
(**A**) Typical layered structures of MAX Phases, (**B**) Typical layered structures of MXenes, and (**C**) Representative Compositions of MAX Phases and MXenes (reprinted with permission from Ref. [17]. Copyright (2020), Springer Nature).

MXenes are a new type of two-dimensional (2D) material, introduced by Naguib et al. in 2011 [13]. Since their discovery, MXenes have already acquired a significant amount of interest within the diverse advanced technological fields, especially in biosensing platform development. These are commonly prepared by chemically wet etching, Lewis acid molten salt etching, or electrochemical processes targeted at selective etching of the A atomic layers of bulk MAX phases. During the MAX phases, the M_n+1_X_n_ layers, in which *M* is an early transition metal and *X* is carbon and/or nitrogen, are interlocked with atomic layers of an element of an *A*-group (usually one of the elements of the groups 13–16, including Al, Ga, Si, Ge, P, or As (Figure 1C). It has the characteristic MAX structures such as M_2_AX, M_3_AX_2_, and M_4_AX_3_ as shown in Figure 1A. In such layered carbides/nitrides, the bonds of the *M* and the *A* are comparatively weaker than those of the *M* and the *X*, and hence with selective chemical removal of the *A* element, leave the integrity of the M_n+1_X_n_ intact [14]. Such selective etching makes it easier to delaminate individual layers, eventually forming MXene structures (Figure 1B).

The exposed transition metals are usually capped with functional groups, including hydroxyl (-OH), fluoride (-F), and oxide (-O), and the overall chemical formula of the surface is M_n+1_X_n_T_x_, where *T_x_* represents the surface endings [15,16,17] (Figure 1C). These surface ends play a vital role in determining the physicochemical and electronic properties of MXenes, giving them a controllable surface chemistry and increased reactivity that form the basis of their emerging usages in electrochemical and biosensing circuits.

Owing to their atomic-layered structure, MXenes exhibit a range of remarkable properties, including high electrical conductivity, a large specific surface area, excellent solution processability, and outstanding mechanical flexibility. These intrinsic features, in combination with the tunability of their surface chemistry via terminal functional groups, render MXenes highly promising candidates as substrate materials for emerging applications such as wearable sensors, energy storage devices, and electrocatalysis.

The surface terminal groups of MXenes not only influence the electronic density of states (DOS) at the Fermi level but also modulate the intrinsic electronic nature of the material, determining whether it exhibits metallic or semiconducting behavior [18,19,20,21]. This modulation induces notable changes in the work function of MXenes, an essential parameter that can be finely tuned by controlling the surface electron density, thereby optimizing the interfacial interactions between the electrode surface and target analytes in electrochemical sensing applications [17,22,23]. Moreover, the negatively charged surface of MXenes—primarily due to the presence of terminal functional groups such as -OH, -F, and -O, offers favorable adsorption sites for positively charged species, including metal ions like Al^3+^, Ag^+^, and Cu^2+^ [21]. These electronic factors dictated by these terminal groups play a crucial role in the interfacial charge-transfer dynamics at the electrode/electrolyte interface, which is of importance in the sensitivity and response rate of electrochemical sensors. As an example, -O terminals have been demonstrated to improve conductivity and electrostatic interaction with negatively charged target molecules, but -F groups can raise the resistance of charge transfer, which is not beneficial to sensor operation that needs high conductivity [19,24,25].

**Figure 2:**
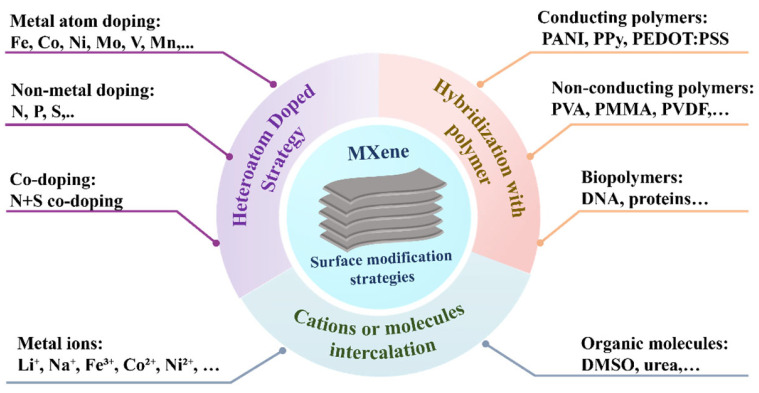
Schematic illustration of different surface modification strategies of MXenes.

Though surface functional groups confer MXenes with a lot of tunability when it comes to electronic and electrochemical properties, there are a number of inherent limitations, such as the ability to recall interlayer restacking, oxidation vulnerability when exposed to outside environments, mechanical inflexibility, and optimum electrochemical behavior [26,27]. To eliminate these negative aspects, it has been highlighted that several surface modification methods have been investigated to form functionalized MXenes with improved characteristics [28], as shown in Figure 2. Among them, one of the common approaches that has been adopted is that of heteroatom doping aimed at enhancing electrical conductivity in the absence of any insulating additive. Another successful technique is cation or organic molecule intercalation, and as a result, it also magnifies the interlayered spacing and does not degrade the electrical conductivity [29,30]. This plan enables the diffusion of ions and the interaction of molecules in the electrochemical. In addition, it tends to be, in general, simple, minimally invasive, and reversible, which leads to the structural stability of MXenes. Also, polymer hybridization is a synergistic blend of mechanical flexibility and versatility. The conduction of the material can be partially decreased by the presence of polymers, but their tunable nature, which makes them conductive, hydrophobic, or biocompatible, makes it possible to advance MXene uses, especially in flexible electronics and wearable biosensors [31,32]. To conclude, surface engineering of MXenes not only increases their physicochemical properties but also opens the capabilities of MXenes as future platforms of smart electronic devices, energy systems, and other highly advanced biosensing technologies.

## 3. Fabrication and Surface Engineering of SPEs

### 3.1. Fabrication of SPEs

**Figure 3:**
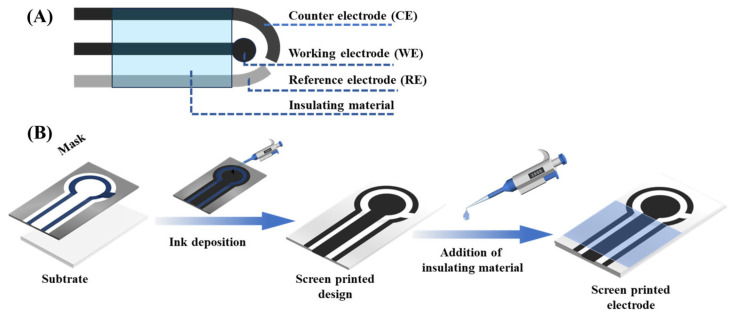
(**A**) A scheme of construction for a printed electrode. (**B**) Schematic representation of the manufacturing process of an SPE.

SPEs have become one of the key technologies in electrochemical biosensing, with their unique set of simplicity, low cost, and dependable analytical qualities. Fabricated through a process analogous to textile screen-printing, conductive inks or functional materials are forced through a patterned mesh screen onto a substrate, forming reproducible and well-defined electrode layouts [33,34,35,36,37]. The method is scalable, assures high manufacturing uniformity, and therefore the SPEs are best suited to the mass-scale production of portable and disposable sensor platforms, especially in the biomedical diagnostic field. In a typical SPE, a three-electrode system, comprising working (WE), counter (CE), and reference (RE) electrodes, is deposited on one planar substrate [38,39] (Figure 3A). The active surface where the electrochemical reactions of the target occur is often the WE, which is made of carbon or gold, or platinum [40,41,42,43,44]. The CE is usually made out of the same material but provides a complete circuit, and the RE, usually Ag/AgCl, provides a constant potential reference. Other than the electrode configuration, the substrate type is also of significant importance since it provides a physical underpinning on which these electrodes are impressed, and it also affects the long-term stability and performance directly. The most frequently employed substrates are ceramics, polymers (e.g., PET, PVC), paper, and textiles [45,46]. Polymers offer the mechanical flexibility required for wearable sensors, whereas ceramic substrates provide durability under harsh conditions [47]. By selecting appropriate electrode materials and substrate designs, SPEs can be adapted for diverse applications ranging from clinical diagnostics to environmental monitoring.

In screen-printing, functional inks are deposited onto a substrate through a patterned mesh screen, typically constructed from fine synthetic fibers (Figure 3B). The structure of the electrodes is characterized by the open spaces of the screen, and the non-opened parts are covered by a non-permeable coating. A squeegee made of rubber then stamps the ink through the mesh, creating clear patterns of electrodes. This procedure is carried out on a case-by-case basis (WE, CE, and RE) with the degree of alignment of paramount importance concerning spatial organization and electrochemical functionality [48,49]. Following the deposition, the printed layers are cured through thermal, UV, or air-drying to cure the ink and increase adhesion [50,51]. Additional functional coating layers could be insulating dielectrics or protective coating (e.g., Nafion) to enhance durability, lowering fouling, or demarcating active electrode regions [52].

### 3.2. Surface Modification and Functionalization of SPEs

For optimal operation, the working electrode of an SPE is commonly modified with a nanomaterial such as MXene [10,11], metal nanoparticles [53,54], graphene [55,56], or carbon nanotubes [57,58,59] to improve its electrochemical performance. The change in this way improves the electrochemical response to target analytes. In printed biosensors, the working electrode cell is further biologicalized with biomolecules like antibodies, enzymes, aptamers, or DNA to allow specific molecular detection. Three major strategies are commonly employed to integrate nanomaterials and biorecognition elements onto printed working electrodes: (i) drop-casting or drop-coating, (ii) electrodeposition, and (iii) formulation of functional inks that can be deposited directly onto the working electrode. The functional layer needs to be highly fixed to the base to avoid dissolution and detachment in the electrolyte in order to retain stability, sensitivity, and reproducibility of the sensor.

Of such techniques, drop-casting and electrodeposition techniques have been large-scale for decades. Zhang et al. [60] designed an acetaminophen (ACOP) and isoniazid (INZ) sensor by drop-casting MXene dispersion on the surface of SPEs. The properties of Ti_3_C_2_T_x_ were traced as the reason behind the excellent performance of this sensor. First, Ti_3_C_2_T_x_ is a titanium-rich material that provides a conductive carbon framework and establishes an efficient electron transport channel. Second, it has an accordion-layered structure that contains numerous active sites and thus, the transfer of electrons through it is quite rapid for the electrocatalytic reaction. Lastly, the negatively charged surface of MXene, containing -F, -OH, and -O functional groups, promotes the adsorption of positively charged analytes such as INZ. In another study, Liu et al. [61] have adopted the use of an in situ electrodeposition method to co-modify SPEs with both AuNPs and MXene to develop a highly sensitive electrochemical biosensor capable of detecting the biomarkers of infectious disease. The flower-shaped nanostructure of AuNPs and MXene increased both sensitivity and specificity through a higher surface area and the rate of transfer of electrons in the nanoparticle, hence improving the rate of electron transfer. These findings indicated that strategies for modifying the SPE are effective in improving sensor performance.

In addition, recent advances in MXene-based functional inks offer a practical approach for fabricating SPEs without additional surface-modification steps. Direct printing of MXene enables a more uniform active layer and improves the reproducibility of SPEs, which is important for biosensing applications. Wu et al. [62] developed aqueous MXene/xanthan gum hybrid inks with adjustable viscosity, good printability, and long-term stability. Screen-printed MXene films prepared from these inks reached a conductivity of 4.8 × 10^4^ S·m^−1^, indicating a compact and continuous printed layer. Although this work did not focus on biosensors, the electrical and structural properties achieved are relevant for SPE fabrication, particularly for electrodes requiring high conductivity to support biomolecule immobilization and stable electrochemical responses. Overall, MXene functional inks represent a promising avenue for the development of next-generation printed electronics and bioanalytical sensing platforms.

## 4. Biomedical Applications of MXene-Based SPE Sensors

Various MXene-based SPE sensors have been designed so far to detect a variety of biomarkers, among them metabolites, neurotransmitters, proteins, and nucleic acids in biological fluids, such as sweat, urine, and serum. Numerous surface modification strategies have been employed to enhance the electrochemical performance of these sensors and mitigate biofouling effects. Table 1 summarizes the analysis of their surface engineering method, the analyte, and analytical and performance characteristics. Several representative examples of MXene-based SPE sensors are discussed in the following sections.

### 4.1. MXene-Based SPE Sensors in Detecting Cancer Biomarkers

**Figure 4:**
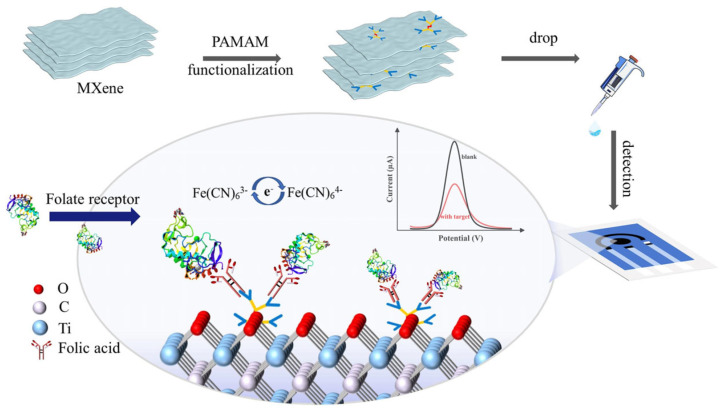
Schematic diagram of the PAMAM@MXene synthesizing procedure and the sensing strategy for detecting FR (reprinted with permission from Ref. [65]. Copyright (2024), Elsevier).

The incorporation of MXenes into SPEs has recently emerged as an effective strategy to improve electrochemical performance for biosensing applications. Owing to their unique two-dimensional metallic conductivity, tunable surface chemistry, and rich terminations (-OH, -O, -F), MXenes provide an ideal platform for rapid electron transfer, strong biomolecule immobilization, and high electroactive surface area. When integrated into SPEs, these features address the inherent limitations of conventional carbon or gold electrodes, such as restricted sensitivity, poor reproducibility, and susceptibility to surface fouling. In addition, the excellent dispersibility and functionalizability of MXenes facilitate their hybridization with polymers, metal nanoparticles, and other nanostructures, thereby creating synergistic effects that amplify the electrochemical signal. Poly(amidoamine) (PAMAM) dendrimers, for example, have been employed to further increase surface loading and enhance probe accessibility due to their controllable size, three-dimensional hyperbranched architecture, and abundant terminal amine groups [75,76]. Jiang et al. [65] developed a Ti_3_C_2_T_x_-PAMAM composite integrated into screen-printed carbon electrodes (SPCEs) for the selective detection of folate receptor (FR), a membrane glycoprotein overexpressed in multiple human malignancies. The PAMAM functionalization prevented MXene restacking, improved surface accessibility, and provided abundant terminal amine groups for biomolecular anchoring. This synergistic configuration combined the high conductivity of MXene with the bio-interactive nature of PAMAM, yielding a broad detection range (10–1000 ng/mL) and a low detection limit (5.6 ng/mL). Furthermore, the sensor exhibited excellent selectivity, reproducibility (RSD < 1.7%), and stability (1.6% signal loss after one week at 4 °C). Overall, this study highlights how rational chemical modification of MXene can go beyond its intrinsic conductivity, enabling control over interlayer spacing, surface chemistry, and biomolecule binding sites. These attributes justify the selection of Ti_3_C_2_T_x_ among other MXenes and underline its potential as a versatile electroactive substrate for high-performance SPE-based biosensors, see Figure 4.

**Figure 5:**
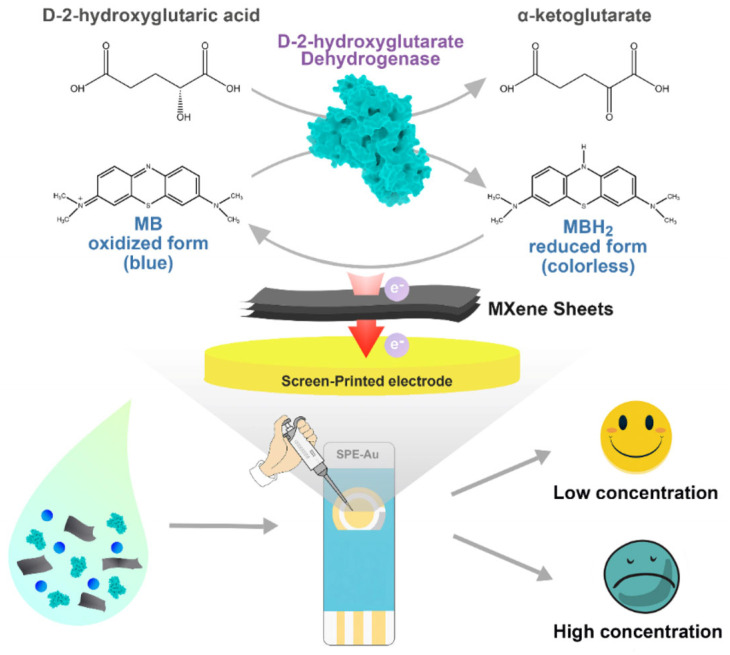
The fabrication and the WE mechanism of the D2HG biosensor (reprinted with permission from Ref. [70]. Copyright (2022), MDPI).

In addition to protein-based targets, MXene-modified SPEs have also been applied for the detection of small molecule metabolites. Wu et al. [70] developed a Ti_3_C_2_T_x_-modified SPCE for the selective detection of D-2-hydroxyglutarate (D2HG), an oncometabolite that accumulates abnormally as a consequence of isocitrate dehydrogenase (IDH) mutations. These mutations, typically occurring at R132 and R172 residues, are closely associated with gliomas, intrahepatic cholangiocarcinoma, chondrosarcoma, and acute myeloid leukemia [77,78,79,80,81]. Owing to its strong association with IDH mutations, D2HG has been recognized as a specific biomarker for both D-2-hydroxyglutaric aciduria and IDH-mutant cancers [82]. In this system, Ti_3_C_2_ MXene served as a conductive and enzyme-compatible substrate with abundant oxygen-containing surface terminations, enabling effective immobilization of *Ralstonia solanacearum* D-2-hydroxyglutarate dehydrogenase (RsD2HGDH) and promoting direct electron transfer through methylene blue (MB) without additional cofactors. The bioelectrocatalytic oxidation of D2HG to α-ketoglutarate (KG) generated a current response linearly proportional to D2HG concentration (0.5–120 μM). The sensor was very sensitive (22.26 μAmM^−1^cm^−2^) and exhibited a low detection limit (0.1 μM). Their outcomes were explained by the fact that Ti_3_C_2_T_x_ displayed a high conductivity and hydrophilic surface chemistry, and therefore, the enhancement of electron transport and enzyme activity with respect to the use of conventional carbon electrodes. The values of 99.56–106.83% and 97.30–102.47% in recovery tests in fetal bovine serum and artificial urine, respectively, indicated that the sensor was feasible in detecting the presence of metabolites in biological fluids. This study emphasizes the performance of MXene to enhance the ability of electron-transfer, stability of enzymes, and the overall performance of the enzyme, in addition to the current challenges in the large-scale production of recombinant enzymes and in incorporating the systems into POC diagnostics, see Figure 5.

As of late, it has been shown that MXene-modified SPEs have strong potential and can be used in nucleic acid diagnostics, especially when paired with biological amplification techniques like CRISPR and duplex-specific nuclease (DSN) systems. The fusion between CRISPR-Cas12a and electrochemical sensor systems (E-CRISPR) has received considerable interest due to its high sensitivity, quantitative response, and the ability to be used with POC testing. H. Duan et al. [73] reported a highly stable and ultrasensitive E-CRISPR platform based on a SPCE modified with an AuNP-decorated Ti_3_C_2_ composite. The AuNPs on top of the MXene nanosheets offered large amounts of binding of the ssDNA through Au–S binding, avoiding the necessity to use large amounts of gold as substrates. There was direct quantification of unamplified human papillomavirus (HPV-16 and HPV-18) DNA in a wide range (10 pM–500 nM) using this platform. The synergistic relationships between AuNPs and MXene enhanced the electron transfer kinetics and antifouling properties, developing a multifaceted approach of MXene-molecule-aided CRISPR electrochemical biosensing. Similarly, M. Mohammadniaei et al. [74] introduced a synergistic amplification system to combine MXene-based electrochemical enhancement with DSN-assisted target recycling to detect the multiplexed cancer biomarker microRNA (miR-21 and miR-141) in plasma. They used a screen-printed gold electrode (SPGE) with tan incorporation of Ti_3_C_2_T_x_ nanosheets sampled with 5 nm Au nanoparticles to create a hierarchical structure, AuNP@MXene/Au. A hybrid interface offered tremendous active sites and surface area to immobilize ssDNA, which led to a fourfold enhanced electrochemical signal compared to the traditional AuNP/Au electrodes. This was explained by high charge mobility, a large specific surface area, and high grades of Au-thiol affinity of MXene, which together facilitated effective electron transfer as well as antifouling stability in complicated biological settings. The biosensor had a limit of detection of 204 aM and 138 aM of miR-21 and miR-141, respectively, in a large linear range (500 aM to 50 nM), and made it possible to process large numbers of clinical samples in parallel by incorporating a 96-well format. The enhanced biomolecular immobilization and electron transfer of MXene nanostructures are due to their superior conductivity, high surface functionality, and antifouling properties, which can be used to combine with CRISPR and DSN-based schemes of amplifying molecular diagnostics next-generation in portable devices.

### 4.2. MXene-Based SPE Sensors for Pathogen Detection

**Figure 6:**
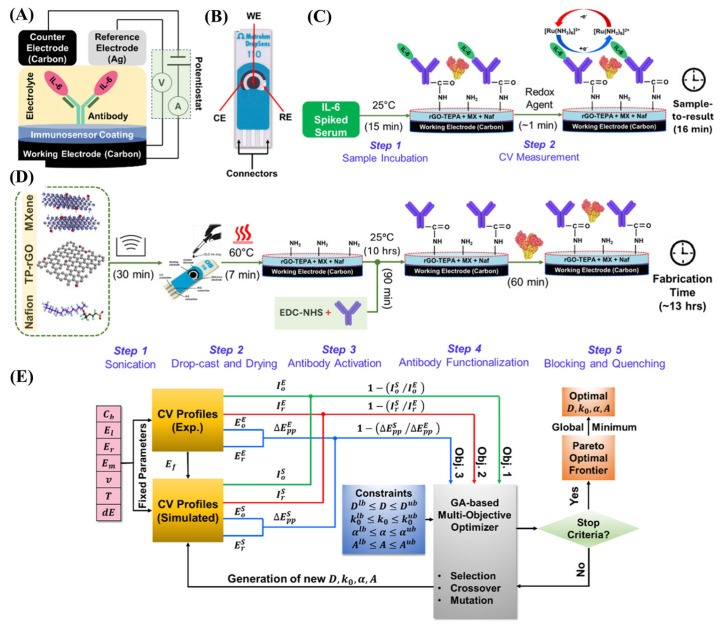
Schematic illustration of IL−6 electrochemical biosensing platforms: (**A**) schematic illustration of the three-electrode sensing mechanism; (**B**) Metrohm DropSens SPCEs with WE, CE, and RE; (**C**) label-free detection strategy for IL−6 in human serum; (**D**) Stepwise fabrication process of the biosensor; (**E**) Mechanistic model integrated with a GA for extracting unknown electrochemical parameters to elucidate the biosensor’s response. (Reprinted with permission from Ref. [64]. Copyright (2025), ACS).

In addition to nucleic acid and small-molecule detection, MXene-amended SPEs have also been utilized to electrochemically detect disease-related proteins. To detect the amount of the highly sensitive proinflammatory cytokine interleukin-6 (IL-6), Gupta et al. [64] fabricated a rapid, label-free, and sensitive electrochemical biosensor (Figure 6A,B). The biosensor was also prepared by applying a Ti_3_C_2_T_x_-based nanocomposite of tetraethylene pentaamine-functionalized reduced graphene oxide (TEPA-rGO) and Nafion (Figure 6D). TEPA-rGO was used in covalent biomolecule binding, especially antibodies, while Nafion, a sulfonated fluoropolymer, improved membrane stability and antifouling performance. The sensor achieved a detection limit in the single-digit pg/mL range with a broad linear response from 3 to 1000 pg/mL, requiring only 5 μL of serum. It displayed extreme selectivity against typical interferents, was stable with a maximum life of one month, and provided a 15-min readout (Figure 6C), which was approximately 12 times fasterthan standard ELISA with the same sensitivity.

Furthermore, a thin-layer diffusion model based on a genetic algorithm was applied to optimize key electrochemical parameters, including the diffusion coefficient, rate constant, and charge transfer coefficient (Figure 6E). This computational approach provided valuable insights into charge-transport dynamics and established a framework for the rational design of high-performance biosensors.

Expanding beyond single-analyte detection, Zhang et al. [69] developed a dual-channel microfluidic electrochemical immunosensor designed for early periodontitis diagnosis and monitoring through simultaneous measurement of interleukin-1β (IL-1β) and matrix metalloproteinase-8 (MMP-8) in saliva. The dual-channel microfluidic chip, featuring two SPEs modified with an IrOx/Ti_3_C_2_T_x_ nanocomposite, reduces reagent use and operational complexity while providing reliable, interference-free quantification of both biomarkers. This compact sensor is suitable for POC testing. Enhanced by IrOx nanotubes and MXene nanosheets, the sensor delivers low detection limits (0.014 ng/mL for IL-1β, 0.13 ng/mL for MMP-8) and wide linear ranges, ensuring high selectivity and accuracy in both artificial and clinical saliva. Most importantly, dual biomarker detection enables more precise disease severity discrimination than single-biomarker approaches. This work demonstrates that dual-channel electrochemical biosensors can significantly advance POC testing for periodontal diagnostics by enabling earlier and more accurate detection. Further validation in larger and more diverse clinical cohorts, as well as long-term stability assessments, is needed before routine application.

In another study, Liu et al. [61] reported a multichannel electrochemical immunosensor enabling simultaneous detection of three major infectious disease biomarkers-HBsAg, anti-HIV antibodies, and anti-TP antibodies. The SPE surfaces were plasma-pretreated and modified via in situ electrodeposition of AuNPs and Ti_3_C_2_T_x_ to improve conductivity and enlarge the active surface area. L-cysteine was immobilized through Au-S bonding, followed by covalent coupling of antibodies via EDC/NHS chemistry. Specific antigen–antibody binding events altered interfacial electron transfer kinetics of the redox probe [Fe(CN)_6_]^4−/3−^, allowing precise quantification of targets in complex serum matrices. The low-cost, disposable SPE platform demonstrated scalability, and allowed us to print 10 × 10 sensors simultaneously. The AuNPs and MXene-SPE sensors achieve broad detection ranges (0.05–1000 ng/mL for HBsAg, 0.35–140 ng/mL for anti-HIV, and 0.25–100 ng/mL for anti-TP) accompanied by low limits of detection (0.01, 0.11, and 0.10 ng/mL, respectively). Applied to standard serum samples, the sensors showcase precision with relative standard deviations consistently below 5.0%, affirming their suitability for complex solution environments. These sensors offer notable advantages, including cost-effectiveness, rapid response, high sensitivity, and specificity, eliminating the necessity for expensive equipment, which opens avenues for the realization of POC testing.

### 4.3. MXene-Based SPE Sensors in Detecting Bodily Metabolites

Nanotechnology has made it possible to achieve significant advancements in the performance of SPEs [83]. Nanosensitivity and selectivity, and stability of operation have been improved by incorporation of nanomaterials like gold nanoparticles (AuNPs) [84,85], two-dimensional materials like MXene [86], and carbon black [87]. Most existing systems, however, still require a benchtop signal-acquisition instrument, which restricts their usage in decentralized or home-based designs. A combination of portable electronics with SPEs represents an important direction for developing practical POC diagnostic platforms.

**Figure 7:**
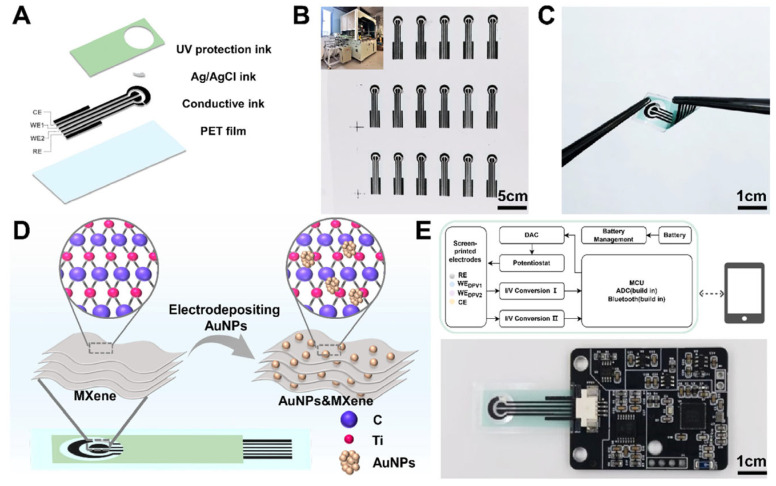
Schematic illustration of the ip-ECS. (**A**) Structure of AuNP/MXene-SPE. (**B**) Large-scale fabrication via automatic screen-printing. (**C**) Individual electrode showing flexibility and wearable applicability. (**D**) Modification of the WE by MXene deposition and AuNP growth. (**E**) Custom electronic module integrated for high-performance electrochemical sensing (reprinted with permission from Ref. [11]. Copyright (2025), ACS Nano).

Chen et al. [11] designed an integrated portable electrochemical sensor (ip-ECS), which comprises MXene-modified SPEs paired with AuNPs and a specific low-power electronic system to measure biomarkers in real-time and wirelessly. PET substrates were prepared by the PET electrode as follows: a series of conductive inks, Ag/AgCl ink, and a UV-protection layer were deposited by using the automatic screen-printing machine (Figure 7A). With automated printing, it was possible to produce large volumes fast and with better reproduction than manually screen-printed products (Figure 7B). The resulting electrodes were small but flexible and thus can be used in portable, wearable, and POC applications (Figure 7C). The working electrodes were also modified using AuNP/MXene nanocomposites (Figure 7D), which caused a boost in electrochemical performance due to the higher number of electroactive sites and charge-transfer efficiency. The electronic module that goes with it was small and consumed minimal power to ensure that it could be used in a portable case (Figure 7E). Leveraging the high conductivity of MXene and the catalytic properties of AuNPs, the system enabled sensitive detection of DA and UA, achieving detection limits of 1.12 μM and 1.11 μM, respectively. The ip-ECS exhibited a good level of concurrence with the conventional electrochemical workstations, which validates the accuracy of the integrated circuitry. The platform was validated using human serum samples. In the case of UA analysis, the system took 2 min to complete the overall measurement process, in contrast to 20 min for biochemical commercial analyzers. In addition, the sensor was found to be linearly quantified and cystatin C (CysC) above 50–5000 ng/mL and closely correlated with latex immunoturbidimetric assays (ρ = 0.9556). Clinical testing in serum from pregnant women further identified elevated Cys C levels in gestational diabetes mellitus (GDM) cases, demonstrating diagnostic relevance. Overall, this work illustrates the potential of combining MXene-modified SPEs with portable electronics to advance practical POC diagnostic systems.

**Figure 8:**
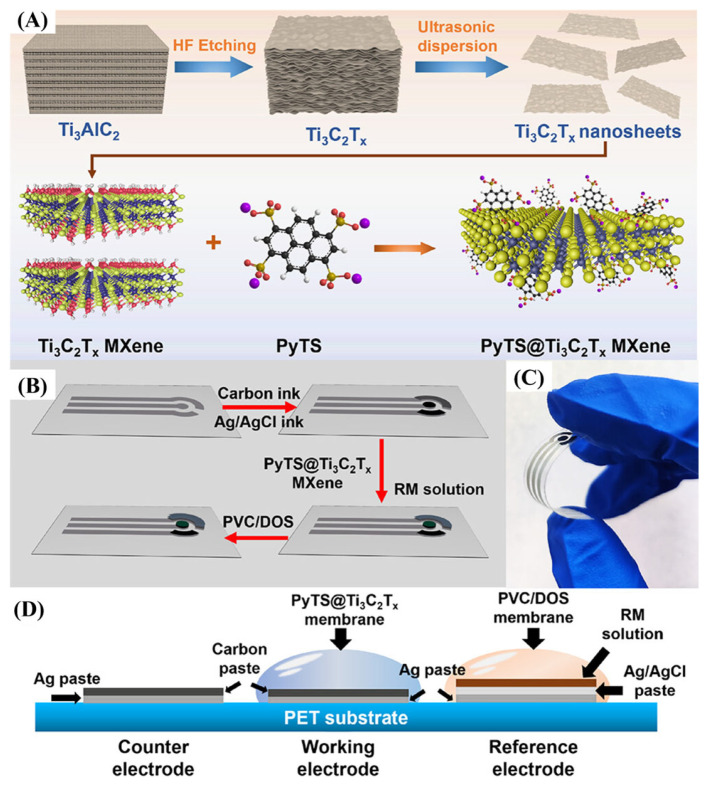
(**A**) Preparation of PyTS@Ti_3_C_2_T_x_ MXene powder; (**B**) schematic of screen-printed pH sensor fabrication; (**C**) photo of the flexible electrode under bending; (**D**) cross-sectional view of the three-electrode configuration. (Reprinted with permission from Ref. [67]. Copyright (2024), American Chemical Society).

Chen et al. [67] reported the development of a wearable electrochemical sensor employing functionalized Ti_3_C_2_T_x_ MXene (PyTS@Ti_3_C_2_T_x_) for real-time UA monitoring via sweat analysis (Figure 8A,B). Functionalization with PyTS significantly enriched the surface redox-active groups of MXene, thereby enhancing its electrocatalytic activity toward UA oxidation. The resulting sensor had high sensitivity over a physiologically relevant range (5–100 μM), detecting 0.48 μM, which is impressive by conventional uricase-based uricase-enzyme sensors. The device was built into a flexible microfluidic platform of sweat collection and integrated with wireless electronics to provide continuous, on-body monitoring of UA during aerobic exercise. Comparison with commercial sweat analyzers of blood UA had shown that there was a great correlation between blood and sweat UA analyses. Even though the absolute sweat concentrations were about a tenth of the blood concentrations, the proportional relationship proved that sweat would be a valid surrogate biofluid in noninvasive biofluid monitoring of UA. In this work, the twofold benefits of MXene-based sensing materials entirely reside in their high conductivity and a large number of redox-active sites, in addition to the fact that it is practically achievable to combine them into flexible, wearable sensors (Figure 8C,D). Taken together, the PyTS@Ti_3_C_2_T_x_-based UA sensor is an important step towards noninvasive, real-time health measurement and a signifier of things to come in terms of MXene-based materials in future wearable electrochemical biosensors.

Lu et al. [63] developed a lactate sensor based on SPCEs modified with Ti_3_C_2_T_x_, polydopamine (PDA), and AgNPs. The accordion-type Ti_3_C_2_T_x_ geometry provides a large surface area and good charge transporting ability, whereas PDA can be further used as a reductant to deposit in situ AgNP and as a binding matrix to stabilize the hybrid structure. Additional conductivity and mechanical strength were provided through the introduction of AgNPs. The sensor recorded a sensitivity of 0.145 μA·mM^−1^ and a detection limit of 0.181 mM (S/N = 3). Persistent testing revealed consistent current outputs at lactate levels of 0–15 mM, with devices kept at 4 °C being able to last up to 7 days, showing practical viability as well as the existence of some constraints relating to longer-term storage. Real-time monitoring of the lactate could be achieved during exercise by integrating into a wearable microfluidic electrochemical platform, which was aided by a bespoke Android application. Their concentrations of lactate measured 6.24 mM with a human sweat validation, which is within physiological ranges. This study highlights the possibility of using Ti_3_C_2_T_x_-AgNP hybrids to achieve both high conductivity and redox functionality in biosensing with considerable reliability, as well as demonstrating the translational potential of biosensors on wearable devices. However, concerns such as storage stability and the effect of complex sweat matrices also need to be considered and researched to exhaust clinical applicability.

The development of SPEs for POC biomarker detection requires addressing biofouling, a major impediment in complex biological matrices. Karuppaiah et al. [10] suggested an antifouling nanocomposite interface (ANcI) based on a three-dimensional hybrid hydrogel comprising conductive Ti_3_C_2_T_x_ to conquer this challenge. Cross-linked carboxymethyl chitosan and sodium carboxymethyl cellulose were used to form the hydrogel network, thus providing the interface with hydrophilicity, film-forming properties, and long-term stability, which also made it an efficient antifouling coating. The addition of Ti_3_C_2_T_x_ nanosheets in this arrangement increased the electron transport and electrochemical responsiveness further, which is better than the conventional 2D nanomaterials. The construction of an estradiol aptasensor was developed based on this design on SPCEs with the ANcI layer that protected SPCEs against antifouling and the presence of gold nanoparticles that immobilized aptamers. The sensor had a clinically relevant detection range (0.1–1000 pg/mL), the detection limit was 0.127 pg/mL, and demonstrated resistance to nonspecific adsorption in human serum and BSA. Primer testing over time observed strong antifouling characteristics, where approximately 90% of the redox signal was maintained through incubating the sample with 1% BSA and approximately 81% with human serum after one month. Altogether, these results demonstrate a logical and efficient approach to biofouling inhibition, which remains a significant hindrance to the clinical deployment of the electrochemical biosensors, by combining the high conductivity of MXene with the antifouling capabilities of hydrogels. Besides its sensitivity and stability, the adaptability of MXene-hydrogel nanocomposites also implies a wider range of applications as surface modifiers, which can extend not only to clinical diagnostics but also to other spheres of food safety and environmental monitoring.

## 5. Challenges and Future Perspectives

SPEs based on MXenes have been reported to exhibit considerable potential for biomedical applications. This possibility can be attributed to the fact that they are highly sensitive, less sensitive to detection as well, and respond quickly to a wide variety of biomarkers. These sensors (as listed in Table 1) have been successfully used to detect (i) cancer-related biomarkers, including D2HG, FR, miRNA and DNA, (ii) pathogenic targets, including IL-1b, MMP-8, IL-6, HBsAg, Anti-HIV, Anti-TP and SARS-CoV-2, and (iii) endogenous metabolites, including uric, serotonin, glucose, lactate, and dopamine. It has been detected in various biological samples, such as serum, urine, and sweat. It has been augmented to improve sensor performance by integrating it with auxiliary nanomaterials.

Notwithstanding these good outcomes, a number of challenges still face them. The first one is that, when MXenes are stored in an inert environment or in a vacuum, they remain stable; however, when they are exposed to oxygen and humidity, especially in aqueous conditions, they are prone to oxidation. Subsequently, this oxidation causes layers of metal oxides to be formed, thus interfering with the electrical and mechanical performance [88]. Therefore, strategy review in the future ought to focus on surface engineering like functionalization and hybridization with auxiliary materials, to improve the stability and reproducibility of complex biological matrices. Second, although the existing wearable SPE sensors allow studying the sweat in real-time, it is still necessary to achieve significant breakthroughs to be able to monitor other biofluids in real-time, including the interstitial fluid or blood. Third, the adoption of sophisticated technologies is also an essential obstacle in clinical translation of MXene-based SPEs. Techniques like microneedle arrays and implantable sensors paired with microfluidics and AI-powered analytics will provide the possibility to enhance real-time, non-invasive, and individual health monitoring [89]. The pattern recognition and predictive diagnostics, which are facilitated with the help of machine learning-simplified data processing, will contribute to the early diagnoses of the disease and a personalized approach to its treatment [90]. Nonetheless, in order to implement successfully, one has to overcome difficulties like signal stability in noisy conditions, standard training data, and energy-saving hardware integration. This smooth integration of these technologies will result in the coming generation of smart MXene-based biosensing systems, which would support the significant application of such systems in remote and point-of-care systems.

## Figures and Tables

**Table 1:** Overview of MXene-based SPE sensors for biomarker detection.

Materials	Analytes	Analysis Method	Stability (Day)	Limit of Detection	Linear Range	[Ref.]
Ti_3_C_2_T_x_/hybridhydrogel	Estradiol	DPV	N.A	1.27 × 10^−4^ ng/mL	0.0001–1 ng/mL	[10]
AuNPs@Ti_3_C_2_T_x_	DA UA	DPV	14	DA: 1.11 μM UA: 1.12 μM	DA: 1–500 μM UA: 1–1000 μM	[11]
Ti_3_C_2_T_x_/AuNPs	Cortisol	DPV	7	0.1 ng/ml	0.5–500 ng/mL	[12]
AuNPsandTi_3_C_2_T_x_	HBsAg Anti-HIV Anti-TP	DPV	28	HBsAg: 0.01 ng/mL Anti-HIV: 0.11 ng/mL Anti-TP: 0.10 ng/mL	HBsAg: 0–1000 ng/mL Anti-HIV: 0–140 ng/mL Anti-TP: 0–100 ng/mL	[60]
NS-TiO_2_ @Ti_3_C_2_T_x_-HG/rGSPE	AA DA UA	Amperometric	21	AA: 0.25 µM DA: 0.1 µM UA: 0.14 µM	AA: 0.1–2200 µM DA: 0.25–400 µM; UA: 0.25–225 µM;	[61]
Ti_3_C_2_T_x_-PDA-AgNPs	Lactate	CV	30	181 µM	5000–25,000 µM	[63]
TEPA-rGO/Ti_3_C_2_T_x_	IL-6	CV	30	0.0021 ng/mL	0.003–1 ng/mL	[64]
PAMAM@Ti_3_C_2_T_x_	FR	DPV	7	5.6 ng/mL	0.001–1 ng/mL	[65]
DNA/Ti_3_C_2_T_x_	SARS-CoV-2	EIS	40	4 × 10^−9^ µM	1 × 10^−7^–1 µM	[66]
PyTS@Ti_3_C_2_T_x_	UA	DPV	N.A	0.48 μM	5–100 μM	[67]
FeVO_4_@Ti_3_C_2_	5-HT	DPV	28	0.00588 µM	0.025–0.75 µM	[68]
IrO_x_/Ti_3_C_2_T_x_	IL-1β MMP-8	DPV	28	IL-1β: 0.014 ng/mL MMP-8: 0.13 ng/mL	IL-1β: 0.1–100 ng/mL MMP-8: 1–200 ng/mL	[69]
RsD2HGDH/Ti_3_C_2_T_x_/MB/AuSPE	D2HG	Amperometry	30	0.1 µM	0.5–120 µM	[70]
Ti_3_C_2_T_x_/SPE	UA Cre	SWV	N.A	UA: 5 µM Cre: 1.2 µM	UA: 30–500 µM Cre: 10–400 µM	[71]
Ti_3_C_2_T_x_-MWCNT	PA TP CF	DPV	30	PA: 0.23 µM TP: 0.57 µM CF: 0.43 µM	PA: 1.0–90.1 µM TP: 2.0–62.0 µM CF: 2.0–90.9 µM	[72]
AuNPs/Ti_3_C_2_	HPV 18- DNA	SWV	60	1.95 × 10^−6^ µM	0.00001–0.5 µM	[73]
AuNP@Ti_3_C_2_T_x_/Au	miR-21 miR-141	DPV	N.A	miR-21: 2.04 × 10^−13^ µM miR-141: 1.38 × 10^−13^ µM	0.5 × 10^−13^–0.05 µM.	[74]

N.A: no value; DA: dopamine; UA: uricacid; AA: ascorbicacid; rGSPE: graphene oxide screen-print electrodes; HG: holey graphene; PDA: polydopamine; TEPA-rGO: tetraethylene pentaamine-functionalized reduced graphene oxide; IL-6: interleukin-6; PAMAM: poly(amidoamine); FR: folate receptor; PyTS: 1,3,6,8-pyrene tetrasulfonic acid sodium salt; 5-HT: serotonin; FeVO_4_: iron vanadate; IrO_x_: iridium oxide nanotubes; IL-1β: interleukin-1β; MMP-8: matrix metalloproteinase-8; *Rs*D2HGDH: D-2-hydroxyglutarate dehydrogenase from *Ralstonia solanacearum*; D2HG: D-2-hydroxyglutaric; MB: methylene blue; AuSPE: gold screen-printed electrode; MWCNTs: multiwalled carbon nanotubes; PA: paracetamol; TP: theophylline; CF: caffeine; Cre: creatinine.

## Data Availability

Data sharing is not applicable.
